# The Parkinson’s disease-associated protein α-synuclein disrupts stress signaling - a possible implication for methamphetamine use?

**DOI:** 10.15698/mic2014.04.137

**Published:** 2014-03-31

**Authors:** Shaoxiao Wang, Stephan N. Witt

**Affiliations:** 1 Department of Biochemistry and Molecular Biology, Louisiana State University Health Sciences Center, USA.; 2 Department of Pharmacology, Toxicology, and Neuroscience, Louisiana State University Health Sciences Center, USA.

**Keywords:** α-synuclein, methamphetamine, Parkinson’s disease, polo-like kinase, signaling

## Abstract

The human neuronal protein α-synuclein (α-syn) has been linked by a plethora of studies as a causative factor in sporadic Parkinson’s disease (PD). To speed the pace of discovery about the biology and pathobiology of α-syn, organisms such as yeast, worms, and flies have been used to investigate the mechanisms by which elevated levels of α-syn are toxic to cells and to screen for drugs and genes that suppress this toxicity. We recently reported [Wang *et al*. Proc. Natl. Acad. Sci. (2012) 109: 16119-16124] that human α-syn, at high expression levels, disrupts stress-activated signal transduction pathways in both yeast and human neuroblastoma cells. Disruption of these signaling pathways ultimately leads to vulnerability to stress and to cell death. Here we discuss how the disruption of cell signaling by α-syn may have relevance to the parkinsonism that is associated with the abuse of the drug methamphetamine (meth).

PD and many other neurodegenerative diseases are due to the accumulation of misfolded and aggregated proteins, and, importantly, some of these species kill cells. In PD, dopaminergic cell death in the mid-brain is believed to be due to the overexpression of or alterations in the neuronal protein α-syn; however, the α-syn species—monomer, tetramer, oligomer, or prion—that kills cells is not yet known. The function of α-syn, which is a non-essential protein, appears to be to regulate synaptic vesicle release from presynaptic membranes.

We recently reported that α-syn is toxic to yeast cells at elevated levels because it competitively inhibits the phosphorylation of endogenous substrates of the essential polo-like kinase Cdc5. Cdc5 controls cytokinesis and stress signaling by controlling the activity of the GTPase Rho1. We found that α-syn blocks Cdc5 from associating with and activating Rho1 GEFs and/or Rho1 GAPs, which reduces the total cellular level of GTP-Rho1 and inhibits the transcription of stress-related genes in the nucleus (specifically, the cell wall integrity pathway genes) (Fig. 1A). Extending the work to human cells, we showed that elevated levels of α-syn also inhibit related stress-activated protein kinase cascades in human cells that include the mitogen-activated protein kinase p38, c-Jun N-terminal kinase, and extracellular-signal regulated kinases 1 and 2 pathways. Inhibition in human cells requires both overexpression of α-syn and high temperatures.

**Figure 1:**
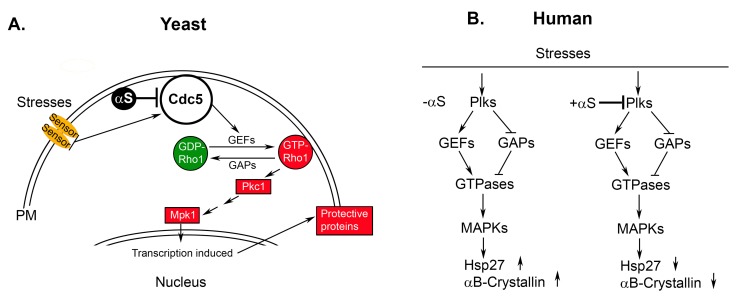
FIGURE 1: α-Syn disrupts cell signaling. **(A)** Model for how α-syn disrupts the cell wall integrity pathway in yeast. This pathway is a mitogen-activated protein kinase pathway that is similar to the p38 stress-activated pathway in humans. **(B)** Model for how α-syn disrupts the p38 stress-activated signaling pathway in humans. In **(A)** and **(B)**, α-syn inhibits the upstream polo-like kinase if and only if one of the following two conditions is met: (i) α-syn is expressed at high levels or (ii) cells are subjected to high temperatures (> 40°C). If conditions (i) and (ii) are met, then inhibition of stress signaling could be very robust. αS, α-synuclein; GEF, guanine exchange factor; GAP, GTPase-activating protein; MAPK, mitogen-activated protein kinase; Mpk1, yeast MAP kinase; Pkc1, yeast protein kinase C; Plks, human polo-like kinase; arrow up indicates increased expression and/or increased activity; arrow down indicates decreased expression and/or decreased activity.

Cells respond to different stresses via two signal transduction pathways. First, heat shock factors (HSFs) are transcription factors that enter the nucleus in response to the accumulation of unfolded proteins that typically occurs when cells are subjected to heat or other stresses. HSFs induce the transcription of genes that contain heat shock elements. The induced proteins, dubbed heat shock proteins (Hsps) or molecular chaperones, protect cells from the unfolded proteins. Second, stress-activated signal transduction pathways, such as the pathway containing the mitogen-activated protein kinase p38, can also be stimulated. Stimuli not only can trigger this pathway to induce the expression of the small heat shock proteins αB-crystallin and Hsp27, the kinases of this pathway also regulate the activities of αB-crystallin and Hsp27 via phosphorylation. These two chaperones stabilize unfolded proteins and inhibit protein aggregation.

We propose that our findings from yeast and human cells about α-syn and stress signaling may shed light on why the risk of PD increases significantly with the abuse of the drug methamphetamine (meth). Meth addiction is a worldwide scourge, and meth addicts have twice the risk of developing PD compared to age-matched controls. Why do meth addicts have an increased risk of PD? Meth has multiple physiological effects on mammals. To name a few, meth inhibits dopamine reuptake from synapses, and generates intense hyperthermia in the brain.

Our hypothesis is that meth, because it induces hyperthermia, drives α-syn toxicity. The temperature in the mid-brain region can exceed 41°C upon ingestion of meth. At such a high temperature, unfolded proteins will accumulate and damage neurons. Dopaminergic neurons, which highly express α-syn, may be particularly susceptible to hyperthermia because of the ability of α-syn to partially inhibit stress-activated protein kinase cascade at elevated temperatures (Fig. 1B) [Wang *et al*. Proc. Natl. Acad. Sci. (2012) 109: 16119-16124]. Specifically, given that p38 pathway regulates the activity of αB-crystallin and Hsp27, if α-syn inhibits p38, this could block the activation of these key chaperones and thus prevent cells from responding to unfolded proteins in the brain that form due to meth-induced hyperthermia. There are at least two ways to test this model. First, the levels and/or activities of both αB-crystallin and Hsp27 should be lower in the dopaminergic neurons of meth users compared to aged-matched controls (Fig. 1B). Second, if meth-induced hyperthermia increases α-syn toxicity, then other drugs that cause hyperthermia in the brain should also increase α-syn toxicity and the risk of PD.

To summarize, in our model, meth molecules do not disrupt kinases in the stress-activated signaling pathways. Instead, the problem lies in the excessive heat induced by meth in combination with α-syn, especially at elevated levels of this protein. During the repeated bouts of excessive heat in the brain, induced by meth, α-syn inhibits stress signaling, which prevents cells from rectifying the damage from the heat, and, over time, this causes neurodegeneration.

